# Health systems research in the time of health system reform in India: a review

**DOI:** 10.1186/1478-4505-12-37

**Published:** 2014-08-09

**Authors:** Krishna D Rao, Radhika Arora, Abdul Ghaffar

**Affiliations:** 1 Health Systems Program, Department of International Health, Johns Hopkins University, 615 N. Wolfe Street, Baltimore, Maryland 21205, USA; 2 Public Health Foundation of India, ISID Campus, Vasant Kunj Institutional Area, Vasant Kunj, New Delhi 110070, India; 3 Alliance for Health Policy and Systems Research, World Health Organization, Avenue Appia 20, 1211 Geneva 27, Switzerland

## Abstract

**Background:**

Research on health systems is an important contributor to improving health system performance. Importantly, research on program and policy implementation can also create a culture of public accountability. In the last decade, significant health system reforms have been implemented in India. These include strengthening the public sector health system through the National Rural Health Mission (NRHM), and expansion of government-sponsored insurance schemes for the poor. This paper provides a situation analysis of health systems research during the reform period.

**Methods:**

We reviewed 9,477 publications between 2005 and 2013 in two online databases, PubMed and IndMED. Articles were classified according to the WHO classification of health systems building blocks.

**Results:**

Our findings indicate the number of publications on health systems progressively increased every year from 92 in 2006 to 314 in 2012. The majority of papers were on service delivery (40%), with fewer on information (16%), medical technology and vaccines (15%), human resources (11%), governance (5%), and financing (8%). Around 70% of articles were lead by an author based in India, the majority by authors located in only four states. Several states, particularly in eastern and northeastern India, did not have a single paper published by a lead author located in a local institution. Moreover, many of these states were not the subject of a single published paper. Further, a few select institutions produced the bulk of research. Of the foreign author lead papers, 77% came from five countries (USA, UK, Canada, Australia, and Switzerland).

**Conclusions:**

The growth of published research during the reform period in India is a positive development. However, bulk of this research is produced in a few states and by a few select institutions Further strengthening health systems research requires attention to neglected health systems domains like human resources, financing, and governance. Importantly, research capacity needs to be strengthened in states and institutions that have a scarcity of researchers, as well as states that have been the focus of little research. While more funding for health systems research is required, this funding needs to be targeted at deficient health systems domains, geographical areas, and institutions.

## Background

Research on health systems is an important contributor to improving health system performance [1]. Such research has informed on the state of country health systems, generated evidence for improving health services, particularly for the poor, and guided health policy [2-4]. In particular, research on program implementation and policy also has the important function of creating a culture of public accountability. In several countries that have committed to reforms for universal health care, research on health systems has played an important role in guiding reforms and monitoring progress [1,5]. In this, the health systems research community plays a critical role as observers of health system reform efforts.

In the last 10 years there have been two major developments in India’s health system. The first was the launch of the National Rural Health Mission (NRHM) (2005–2013) to strengthen the public sector health system, particularly for primary care [6]. Through the NRHM large investments have been made in strengthening public sector health systems, establishing a national conditional cash transfer program for institutional deliveries, as well as a community health worker program. The second has been the introduction, since 2008, of government-sponsored insurance schemes covering hospital care for the poor [7]. Both state and central insurance schemes are present and it is estimated that, by 2015, 50% of India’s population will be covered by government insurance schemes [7]. These reforms have the potential of significantly altering health systems in India, as well as to move the country closer to Universal Health Care. Importantly, these reforms have been accompanied by a growing interest in health systems research. For one, the National Health Systems Resource Centers, and its affiliate State Health Resource Centers, were established under NRHM to provide research and technical assistance to the Ministry of Health and Family Welfare, and NRHM. In 2007, two years after the start of the NRHM, the Department of Health Research was established with a mandate to improve research both in the public and clinical aspects of health care. The Ministry also held two consultations with the health systems research community in 2013 to explore ways of improving the interface between health policy and research. The government-sponsored insurance schemes too are beginning to give importance on research. For instance, the Aarogyasri Health Care Trust, which implements the Rajiv Aarogyasri health insurance scheme in Andhra Pradesh, has created an embedded research unit.

Like in many low- and middle-income countries, research on health systems in India has relatively recent beginnings [1]. The broad trends in this field have generally followed the shifting patterns of both national and global health policies. One of the first comprehensive assessments of the state of the country’s health and health system was produced by the Bhore Committee (1946) report, which also provided the blueprint for India’s public sector health system [8]. The period following the Bhore Committee report saw an expansion of the public sector health system; subsequent committee reports, as well as research on health systems, mirrored the government’s preoccupation with expanding coverage of health services, including, human resources. Studies on health financing began to emerge in the 1960s and its subsequent growth was facilitated by the availability of national sample surveys. In recent years, research attention in India has expanded to include other health system domains. A review of health system research studies from India between 2001 to 2008 reported finding studies covered all the health system domains of health policy/governance (36%), health services (27%), health financing (25%), human resources/training (14%), medical technology (4%), and information systems (2%) [9]. Interestingly, it appears that the early dominance of health service studies was replaced by health policy studies.

This paper provides a situation analysis of health systems research in India between 2005 and 2013. This period covers the implementation period of the two major health system reforms – NRHM and the government-sponsored health insurance schemes. Of interest is to analyze the research response to these important health system reforms. We reviewed published articles on health systems research during the period 2005–2013 and examined temporal trends in publications, the health system areas covered in this research, the characteristics of the researchers, and the geographical areas covered in published health systems research.

## Methods

Health systems research in the context of this review refers to published journal articles and systematic reviews. Two online databases, PubMed and IndMED, were used to search for relevant papers [10,11]. PubMed is a free online resource (database) maintained by the National Center for Biotechnology at the U.S. National Library of Medicine. IndMED is an online database, maintained by the National Informatics Centre and the Indian Council of Medical Research.

The National Library of Medicine’s Medical Subject Headings (MeSH) terms relevant to the WHO health system building blocks were used to search both databases [12]. The following keywords were selected: ‘health service delivery’ (delivery of health care; health services research; quality assurance, health care; quality of health care); ‘health workforce’ (health manpower; health personnel; health services); ‘health information’ (health information management; health information systems); ‘essential medicines’; ‘health financing’ (economics; organizations); ‘leadership and governance’ (policy; social control, formal; government regulation).

A total of 9,477 abstracts/papers published between 2005 and 2013 were obtained from searches on PubMed (6,902) and IndMED (2,575). The cut-off years mark the duration of the NRHM. Not included were papers that could not be classified as health systems research, such as those on epidemiological studies. Further, comments, news articles, withdrawals, and errata were excluded from the study. PubMed advanced filters were used to include ‘Journal Articles’ and ‘Systematic Reviews’.

A total of 1,617 papers out of 9,477 met our inclusion criterion. All analysis is based on the 1,617 papers. Each included paper was reviewed and classified in separate fields according to i) the state of India that the first author’s affiliated organization is located; ii) country that the first author’s affiliated organization is located; iii) state[s] or region of India where the research was conducted and to which the results were applicable; iv) year of publication; and v) the health systems domain[s] that the article addressed. Health system domains were classified according to WHO Building Blocks as ‘financing’, ‘health workforce’, ‘leadership/governance’, ‘medical products, vaccines and technologies’, ‘information’, and ‘service delivery’ [12]. In cases where a paper addressed two or more domains they were classified as ‘cross-cutting’. Note that in the analysis we assume that the first author is the lead author.

A database was created on Microsoft Excel 2010. The entries in the final database were reviewed to check for errors such as duplications and appropriateness of classification.

## Results

**Figure 1:**
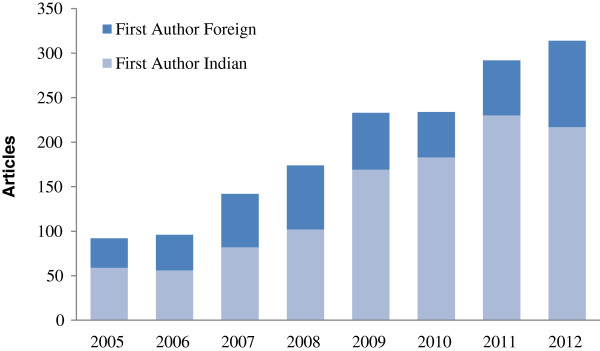
Trend in health systems research publications.

**Figure 2:**
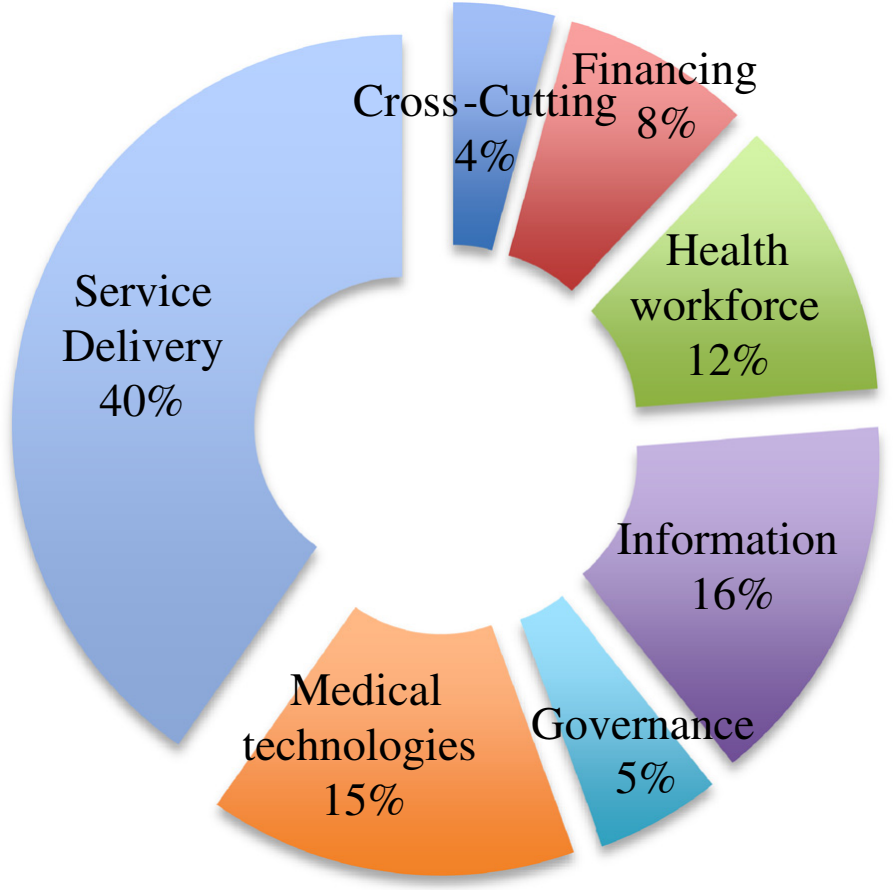
Health system domains covered in publications.

**Figure 3:**
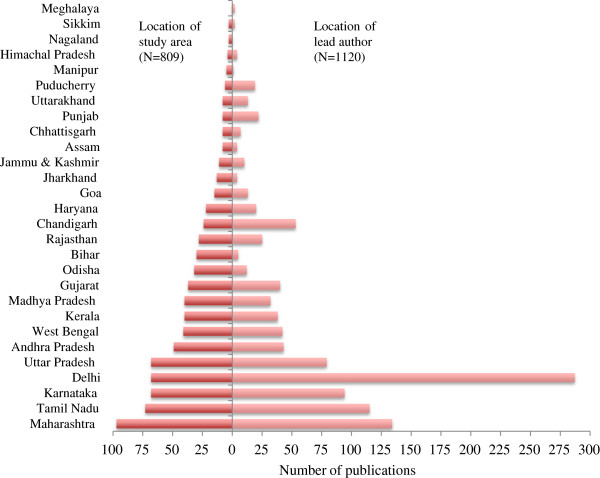
Distribution of publication by states where Indian lead authors and study areas are located.

There has been a consistent increase in published research on health systems between 2005 and 2012 (Figure 1). The increase has been from around 92 publications per year in 2005 to 314 per year in 2012, more than a three-fold increase. This growth in health systems research publications is through papers led by authors in an Indian institution. In 2005, there were only 59 publications where the lead author was based at an Indian institution and by 2012 this had increased to 217, nearly a four-fold increase. Publications by lead authors in a foreign institution also increased; between 2005 and 2012 these increased from 33 to 97, almost a three-fold growth. Over this period, the proportion of publications by authors at Indian institutions increased slightly from 64% to 69%.Figure 2 presents the different health system domains covered by publications in the NRHM period. These domains correspond to the WHO health system building blocks of service delivery, health workforce, information, medical products/technology, and governance. Expectedly, the majority (40%) of publications fall into the exclusive domain of service delivery, followed by information (16%), medical products/technology (15%), health workforce (12%), financing (8%), and governance (5%). Around 4% of the publications addressed multiple health system domains.Figure 3 shows the states in which the lead authors of the reviewed publications were located, as well as the states that were studied. Overall, states that produced more publications were also studied more in the health systems literature reviewed. The state of Delhi (287) had the highest and Manipur (1) the lowest number of publications by locally located lead authors. Overall, the majority of health systems publications were from lead authors located in a few states; for instance 56% of the publications were from the four states (Delhi, Maharashtra, Tamil Nadu, and Karnataka). Moreover, the states of Chhattisgarh, Bihar, Assam, Himachal Pradesh, Jharkhand, Meghalaya, Sikkim, and Manipur had less than one publication a year during the review period (2005–2013). Several states – Nagaland, Arunachal Pradesh, Mizoram, Tripura – had no publications on health systems research in our search results.Figure 3 also shows the states that were studied in the publications reviewed. A total of 809 publications identified a state(s) where the research was conducted. Maharashtra (97) was the focus of the largest number of publications and Sikkim (3) had the least. Six states – Maharashtra, Tamil Nadu, Karnataka, Delhi, Uttar Pradesh, and Andhra Pradesh – were the focus of a little over half the publications in which the state (under study) was identified. States like Assam, Chhattisgarh, Punjab, Uttarakhand, Manipur, Himachal Pradesh, Nagaland, and Sikkim had, on average, one or fewer publications per year during the period of this review. Moreover, the states of Arunachal Pradesh, Mizoram, Tripura, and Meghalaya were not the subject of any publication during the period of review.

**Figure 4:**
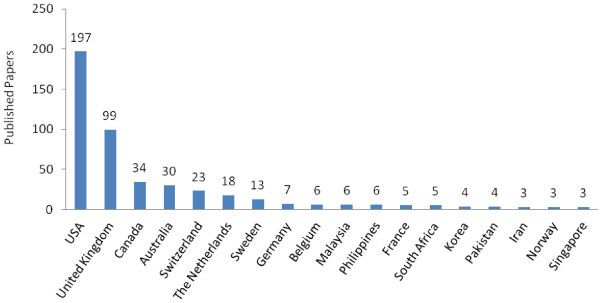
Location of international lead authors.

A large number of institutions contributed to publications on health systems research. For instance, in the states of Delhi, Maharashtra, Tamil Nadu, and Karnataka, which were responsible for 630 or over half of the Indian author lead publications, a total of 219 individual institutions contributed to the publications produced in these states during the review period. However, the productivity of these institutions varied widely. While on average there were only 2.89 publications per institution during the review period, certain institutions were considerably more productive. For instance, with 50 publications, the All India Institute of Medical Sciences in Delhi was by far the most productive nationally. In general, a few select institutions were responsible for a substantial portion of the publications produced by in these four states.Publications led by authors located in institutions outside India are described in Figure 4. The United States contributed the most publications (197). Around 60% of the foreign author led publications were from authors based in the United States and United Kingdom.

## Discussion

### More research needed in the time of reform

This study reviewed published research on health systems during a period of health systems reforms in India. Between 2005 and 2013, the period of this review, two major reforms – the NRHM and government-sponsored insurance – were launched and implemented nationally. Findings from this study indicate that there has been a progressive growth in health systems research publications on India during this reform period. Moreover, this growth is fuelled by publications led by Indian authors. This growth in publications is in keeping with global trends, however, the increase in publications by authors based in local institutions, rather than outside the country, makes India’s experience somewhat unique. [4]. The growth in publications speaks of the enthusiastic response of the research community to the ongoing reform efforts in India. Because local researchers have largely led this growth, it suggests increased local interest and a capacity for health systems research that caters to local needs.

Research on service delivery was by far the most popular health system domain covered by the publications reviewed. This is not surprising given the traditional emphasis on service delivery by policy decision makers as well as researchers. However, what is surprising is the research neglect of certain health system domains like governance, human resources, and health financing. The lack of research in these areas has reduced the comprehensiveness of health systems research in India. Moreover, these domains are major areas of focus within NRHM and the government-sponsored insurance schemes; the scarcity of research in these areas leaves a knowledge gap of the functioning of these aspects of the health system reforms. Importantly, the scarcity of research in these domains reduces the potential contribution of research to improving health system performance, and accountability.

Two important findings emerged in the review regarding the location of researchers and research. For one, researchers located in few states produced most of the publications lead by Indian authors. This is not altogether surprising, given that research institutions tend to be clustered in a few major cities of the country. However, what is of particular concern is that in several states (Chhattisgarh, Bihar, Assam, Himachal Pradesh, Jharkhand, Meghalaya, Sikkim, Manipur, Nagaland, Arunachal Pradesh, Mizoram, and Tripura) there were few or no publications produced by resident researchers during the reform period. To put this into perspective, the area and population covered by any of these states would make them respectably sized countries. This indicates that several states in India have little health systems research capacity, despite being home to large populations. Further, it highlights the skewed distribution of health system research capacity in the country. Many of these low research capacity states are also poor performers in health.

Health systems research in the reform period has focused only on a few states. Notably, several states (Assam, Chattishgarh, Punjab, Uttarakhand, Manipur, Himachal Pradesh, Nagaland, Sikkim, Arunachal Pradesh, Mizoram, Tripura, and Meghalaya) had few or no published studies on their health systems during the reform period. This despite substantial NRHM and government insurance funds flowing to these states, their health systems catering to the need of large populations, and some of them identified as high focus states by NRHM. The lack of health system studies in these states, unfortunately, deprives them of independent assessments of their health system’s performance, and the demands of accountability that such research generates. Finally, states that produced more publications were also studied more in the literature. This suggests the importance of strengthening state level capacity in health systems research.

Our findings indicate that a large number of institutions in India contributed to published health systems research in the review period. This is an encouraging development and is suggestive of the institutional spread of health systems research. However, it appears that only a few select institutions have been responsible for a substantial proportion of the publications generated. This difference in institutional productivity is likely tied to success with securing research funding and also the number of researchers employed in the institution. Taken together with the earlier findings, the picture that emerges suggests that the bulk of health systems research in India is produced in a few states and by a few select institutions.

The deficiency in health systems research in India, therefore, occurs in four areas, namely the health system domains covered, the capacity of certain states to produce research, the states that have been studied in this research, and that only a select number of institutions are producing this research. These deficiencies have implications for the focus and funding of health systems research in India. Clearly, more and sustained funding to further encourage the growth of health systems research as the country expands on its reform program is important. However, equally important is that this funding be so directed that it promotes the development of research capacity and research in states and institutions that have a scarcity of researchers and research. Funding also needs to encourage the expansion of research to understudied domains such as governance, human resources, and health financing. This will not only make the health systems research produced in India more comprehensive in content and geography, but will also strengthen its accountability function in these areas.

This study has several limitations. First, we searched the database for publications on the health system building blocks and not directly on the two reform programs. Consequently, the publications selected need not have been intended to specifically study these reform programs. However, because strengthening health systems is a focus of their reform activities, and these programs influence, and in turn are influenced by, the health system context they operate in, a broader search of the literature covering the six health system building blocks is more appropriate. Second, by limiting our publication dates to the period 2005–2013, it is possible that we included articles that were written before the reforms began (but were published in this period) and excluded those that are set in this period but not published in this time. To the extent possible we have tried to minimize the former issue by checking the period the study was conducted. Third, one Indian journal, *Economic and Political Weekly*, that carries articles of health systems research is only partially indexed in PubMed and therefore our search would not have picked up all articles on health systems research published in this periodical. However, it is not expected that this would have changed the broad results. Fourth, there is a considerable grey literature on health systems that is excluded from the purview of this study since only peer reviewed publications available in PubMed and IndMED were included. Consequently, this review includes only a portion of health systems research published during the NRHM period. However, we believe that we have captured the peer reviewed published literature by using both PubMed and IndMED. Further, we expect that the distribution of the grey literature would mirror that of the published literature and so the major findings of this study continue to hold. Moreover, by limiting the analysis to peer reviewed publications, only those studies that have undergone some independent quality control through peer review enter the analysis.

## Conclusions

Research on health systems can be critical to the success of health reforms by providing independent evaluations of performance, identifying areas that need strengthening, and guiding the reform process by making research findings public. In particular, research on health system performance that is publicly available has the important function of creating an environment and culture of accountability. The growth in published studies on health systems during the period of reforms is a positive development in India’s context. However, this review has also highlighted deficiencies – several important health system domains have received little attention, moreover, several states have low research capacity and have been the subject of little health systems research, and only a few select institutions are responsible for the bulk of the research produced. Strengthening health systems research in India not only needs more funding, but funding that is targeted at improving these research deficiencies.

## Abbreviations

NRHM: National Rural Health Mission.

## Competing interests

The authors declare that they have no competing interests.

## Authors’ contributions

KDR, RA, and AG were involved in conceptualizing the paper. KDR and RA contributed to data analysis, paper writing, and editing. AG contributed to writing and editing the paper. KDR, RA, and AG reviewed the paper. All authors read and approved the final manuscript.
